# OASIS/CREB3L1 is a factor that responds to nuclear envelope stress

**DOI:** 10.1038/s41420-021-00540-x

**Published:** 2021-06-29

**Authors:** Yasunao Kamikawa, Atsushi Saito, Koji Matsuhisa, Masayuki Kaneko, Rie Asada, Yasunori Horikoshi, Satoshi Tashiro, Kazunori Imaizumi

**Affiliations:** 1 Department of Stress Protein Processing, Institute of Biomedical & Health Sciences, Hiroshima University, Hiroshima, Japan; 2 Department of Biochemistry, Institute of Biomedical & Health Sciences, Hiroshima University, Hiroshima, Japan; 3 Department of Pharmacology and Therapeutic Innovation, Nagasaki University Graduate School of Biomedical Sciences, Nagasaki, Japan; 4 Division of Endocrinology, Metabolism & Lipid Research, Washington University School of Medicine in St. Louis, St. Louis, MO USA; 5 Department of Cellular Biology, Research Institute for Radiation Biology and Medicine, Hiroshima University, Hiroshima, Japan

**Keywords:** Cell biology, Biochemistry

## Abstract

The nuclear envelope (NE) safeguards the genome and is pivotal for regulating genome activity as the structural scaffold of higher-order chromatin organization. NE had been thought as the stable during the interphase of cell cycle. However, recent studies have revealed that the NE can be damaged by various stresses such as mechanical stress and cellular senescence. These types of stresses are called NE stress. It has been proposed that NE stress is closely related to cellular dysfunctions such as genome instability and cell death. Here, we found that an endoplasmic reticulum (ER)-resident transmembrane transcription factor, OASIS, accumulates at damaged NE. Notably, the major components of nuclear lamina, Lamin proteins were depleted at the NE where OASIS accumulates. We previously demonstrated that OASIS is cleaved at the membrane domain in response to ER stress. In contrast, OASIS accumulates as the full-length form to damaged NE in response to NE stress. The accumulation to damaged NE is specific for OASIS among OASIS family members. Intriguingly, OASIS colocalizes with the components of linker of nucleoskeleton and cytoskeleton complexes, SUN2 and Nesprin-2 at the damaged NE. OASIS partially colocalizes with BAF, LEM domain proteins, and a component of ESCRT III, which are involved in the repair of ruptured NE. Furthermore, OASIS suppresses DNA damage induced by NE stress and restores nuclear deformation under NE stress conditions. Our findings reveal a novel NE stress response pathway mediated by OASIS.

## Introduction

It had been thought that the nuclear envelope (NE) is stable during interphase [1]. However, accumulating evidence has revealed that NE can be damaged by a variety of cellular stresses called “NE stress”, which lead to the degeneration of NE proteins and disruption of their functional interactions [2–6]. The NE damage is manifested by disruption of the nuclear lamina, including the discontinuous localization of Lamin proteins on the NE and the formation of nuclear blebs accompanied by chromatin herniation [7, 8]. NE rupture often occurs at these regions due to their compromised mechanical strength and results in the loss of compartmentalization between the nucleus and the cytoplasm. NE stress further induces genome instability and cell death [3, 5, 6, 9, 10]. These findings suggest that NE stress perturbs cellular homeostasis and plays crucial roles in the pathogenesis of some diseases caused by dysfunction of NE. Indeed, elevated NE stress has been observed in the cells derived from the patients of Hutchinson Gilford Progeria Syndrome (HGPS), which is caused by the mutation in the gene encoding Lamin A [3].

Recent studies have established that NE rupture is repaired by a series of factors that mediate the reassembly of NE components [11, 12]. A chromatin binding factor BAF functions as a sensor of NE rupture by detecting the cytoplasmic DNA and targets LAP2-emerin-MAN1 (LEM) domain proteins to the ruptured sites [13, 14]. LEM domain proteins further recruit the components of the ESCRT III (endosomal sorting complexes required for transport III) complexes such as CHMP7 [5, 6, 15, 16]. It has been proposed that ESCRT III seals the ruptured NE [5, 6, 15, 16].

OASIS (CREB3L1) is an ER-resident transmembrane transcription factor belonging to the CREB/ATF family [17, 18]. We have demonstrated that OASIS is activated in response to ER stress [19, 20]. The activation of OASIS requires its sequential cleavage mediated by two membrane-bound proteases [21]. The resulting N-terminal region of OASIS, which contains a transactivation domain and a basic leucine zipper (bZIP) type DNA binding domain, functions as a transcription factor.

In this study, we showed that OASIS specifically accumulates at damaged NE and can act in response to NE stress. The discovery of this novel pathway that responds to NE stress is important not only for understanding cellular response to damaged NE, but also for finding clues to develop therapeutic strategies for nuclear envelopathies.

## Results

### OASIS accumulates on damaged NE where nuclear lamina is disrupted

**Fig. 1:**
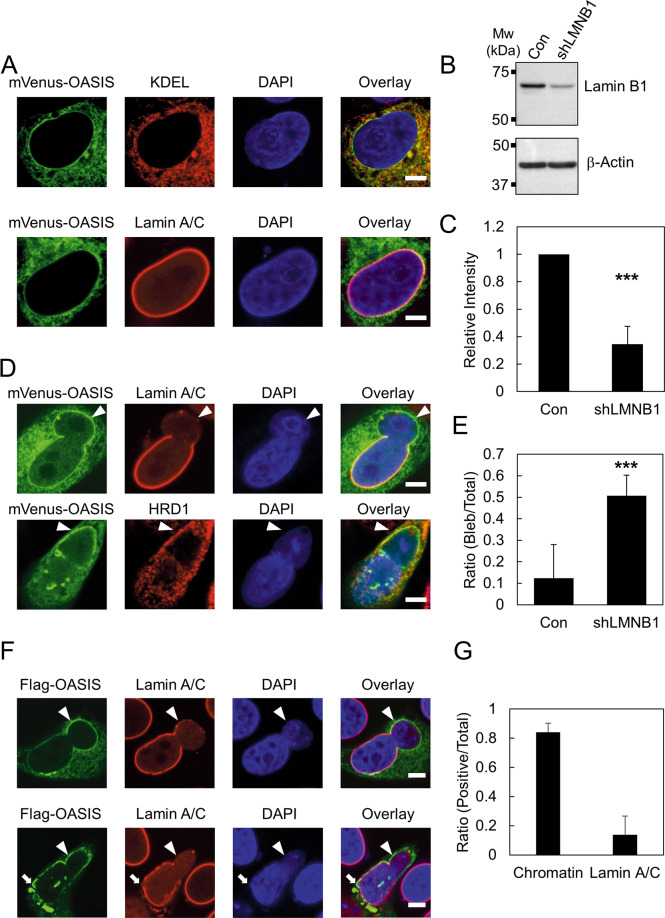
OASIS accumulates on damaged NE where nuclear lamina is disrupted. **A** Immunofluorescence staining analysis of KDEL and Lamin A/C in HeLa cells expressing mVenus-OASIS. **B** Western blotting analysis of HeLa cells stably expressing shRNA against Lamin B1 (shLMNB1). The protein level of Lamin B1 was compared to that of control cells (Con). β-Actin was used as a loading control. **C** Quantification of relative protein levels in (B). Bars and error bars represent the mean values and SD from four independent experiments, respectively. The statistical significance of differences was determined using Student’s *t*-test. ***p < 0.005. **D** Immunofluorescence staining analysis of Lamin A/C and HRD1 in shLMNB1 cells expressing mVenus-OASIS. Arrowheads indicate the nuclear blebs where OASIS accumulates. **E** Quantification of the ratios of control (Con) and shLMNB1 cells in which nuclear blebs formed relative to the total cells expressing mVenus-OASIS. Bars and error bars represent the mean values and SD from three independent experiments, respectively. The statistical significance of differences was determined using Fisher’s exact test. ***p < 0.005. *n* = 35 Con, *n* = 46 shLMNB1. **F** Immunofluorescence staining analysis of Flag-OASIS and Lamin A/C in shLMNB1 cells expressing Flag-OASIS. Arrowheads indicate the nuclear bleb where OASIS accumulates and Lamin A/C is depleted. Arrow indicates the discontinuous localization of Lamin A/C and the accumulation of OASIS. **G** Quantification of the ratio of nuclear blebs that include chromatin and that retain Lamin A/C signals. The nuclear blebs revealing the accumulation of mVenus-OASIS were subjected to quantification. Bars and error bars represent the mean values and SD from three independent experiments, respectively. *n* = 32 Chromatin, *n* = 27 Lamin A/C. Scale bars: 5 μm.

OASIS is mainly localized to the ER under normal conditions. Interestingly, OASIS is also enriched in close proximity to the nucleus [19]. To elucidate the detailed subcellular localization of OASIS, the expression vector of the monomeric yellow fluorescent protein mVenus fused to OASIS at its N-terminus (mVenus-OASIS) was transfected into HeLa cells. mVenus-OASIS signals mainly overlapped with those of an ER marker KDEL (Fig. 1A). Furthermore, they partially colocalized with Lamin A/C, the major components of the nuclear lamina (Fig. 1A). The same localization pattern of mVenus-OASIS was observed in U2OS cells (Fig. S1). These results indicate that a certain amount of OASIS localizes to the NE, where it might play an important role.

Previous studies demonstrated that reduced Lamin B1 level results in disorganization of the nuclear lamina and it can be visualized as a discontinuous localization of Lamin A/C or the formation of nuclear blebs accompanied by chromatin herniation [7, 8]. We generated a cell line in which shRNA against Lamin B1 is stably expressed and designated as shLMNB1. We confirmed a reduction of Lamin B1 protein of ~60% in shLMNB1 cells (Fig. 1B, C). To test the subcellular localization of OASIS in shLMNB1 cells, mVenus-OASIS was expressed in shLMNB1 cells. We observed the formation of nuclear blebs in ~20% of shLMNB1 cells. Intriguingly, strong signals of mVenus-OASIS were observed at the periphery of those nuclear blebs (Fig. 1D, E). In contrast, Lamin A/C were almost completely depleted from the periphery of the nuclear blebs where OASIS accumulated (Fig. 1D). These results indicate that OASIS and Lamin A/C have a mutually exclusive localization pattern at the periphery of the nuclear blebs (Fig. 1D). HRD1, an ER membrane-resident protein, did not accumulate on the nuclear blebs where intense signals of mVenus-OASIS were observed (Fig. 1D). Thus, the accumulation of OASIS at the nuclear blebs is not a consequence of wrapping the nuclear blebs in the ER membranes.

We also analyzed the localization of OASIS on the nuclear blebs using the expression vector for OASIS tagged with Flag at its N-terminus (Flag-OASIS). Flag-OASIS also accumulated at the periphery of the nuclear blebs where Lamin A/C were absent, just like mVenus-OASIS (Fig. 1F, G). We observed the signals of DAPI inside these nuclear blebs in 80% of cells (Fig. 1D, F, G). Thus, the majority of these nuclear blebs are accompanied by chromatin herniation. Consistent with previous studies, we found the discontinuous localization of Lamin A/C at the nuclear rim in some of the shLMNB1 cells [7, 8]. Interestingly, Flag-OASIS revealed focal accumulation at those regions where Lamin A/C were absent (Fig. 1F). These results indicate that OASIS specifically accumulates on the damaged NE where the nuclear lamina is disrupted in shLMNB1 cells.

**Fig. 2:**
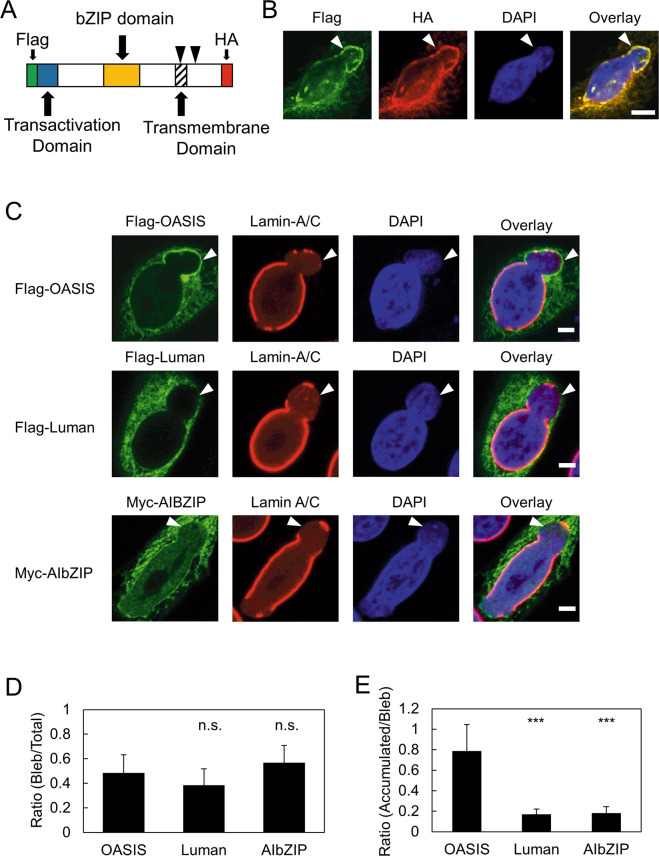
Full-length OASIS specifically accumulates on damaged NE among OASIS family proteins. **A** Schematic representation of the domain structure of OASIS. Arrowheads indicate the cleavage sites of OASIS. **B** Immunofluorescence staining analysis of Flag and HA in shLMNB1 cells expressing Flag-OASIS-HA. Arrowhead indicates a nuclear bleb where OASIS accumulates. **C** Immunofluorescence staining analysis of Flag-OASIS, Flag-Luman, or Myc-AIbZIP in shLMNB1 cells in which nuclear blebs formed. Arrowheads indicate nuclear blebs. **D** Quantification of the ratio of cells in which nuclear blebs formed relative to total Flag-OASIS-, Flag-Luman-, and Myc-AIbZIP-expressing cells. Bars and error bars represent the mean values and SD from three independent experiments. Statistical significance of the difference was determined using Fisher’s exact test. n.s. *p* > 0.05. *n* = 59 OASIS, *n* = 78 Luman, *n* = 55 AIbZIP. **E** Quantification of the ratio of cells in which Flag-OASIS, Flag-Luman, or Myc-AIbZIP accumulate at nuclear blebs relative to total nuclear bleb positive cells expressing the indicated protein. Bars and error bars represent the mean values and SD from three independent experiments. The statistical significance of differences was determined using Fisher’s exact test. n.s. *p* > 0.05. ***p < 0.005. *n* = 30 OASIS, *n* = 31 Luman, *n* = 39 AIbZIP. Scale bars: 5 μm.

Next, we investigated whether the OASIS accumulated at damaged NE is the full-length or cleaved form because OASIS is cleaved at the transmembrane domain in response to ER stress (Fig. 2A) [19–21]. We constructed an expression vector of OASIS tagged with Flag and HA at its N-terminus and C-terminus, respectively (Flag-OASIS-HA) (Fig. 2A). This expression vector was transfected into shLMNB1 cells, followed by immunofluorescent staining. The signals of Flag and HA were almost identical, including at the nuclear blebs (Fig. 2B). Further, we tested the localization of a chimeric protein of which C-terminal transmembrane and luminal domains of OASIS are replaced by those of Luman, another member of OASIS family and its domain structure is similar to that of OASIS [22]. This protein is designated as OL-Chimera hereafter. OL-Chimera is resistant to cleavage as confirmed by western blot and accumulates at the nuclear blebs, suggesting that OASIS accumulates at the damaged NE as full-length form (Fig. S2A, B).

All the OASIS family members share similar domain structures and activation processes [22]. Thus, we examined whether the accumulation on damaged NE is a common feature of OASIS family proteins. Two other members of the OASIS family, Luman tagged with Flag (Flag-Luman) and AIbZIP tagged with Myc (Myc-AIbZIP) at their N-terminus, were expressed in shLMNB1 cells and their localization was investigated. The frequency of nuclear bleb formation was not significantly altered among the cells expressing Flag-OASIS, Flag-Luman, and Myc-AIbZIP (Fig. 2C, D). However, in contrast to Flag-OASIS, Flag-Luman and Myc-AIbZIP hardly accumulated on the nuclear blebs (Fig. 2C, E). These results indicate that OASIS specifically accumulates on damaged NE among the OASIS family proteins.

### Inhibition of proteasome-dependent degradation attenuates the accumulation of OASIS at the damaged NE

**Fig. 3:**
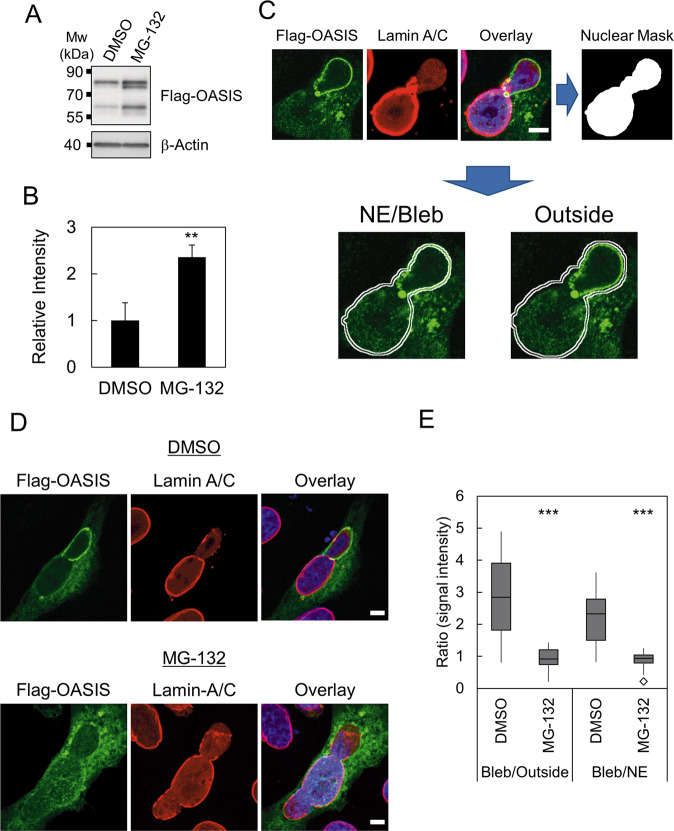
Inhibition of proteasome-dependent degradation attenuates the accumulation of OASIS at the damaged NE. **A** Western blotting analysis of shLMNB1 cells expressing Flag-OASIS treated with DMSO or MG-132. β-Actin was used as a loading control. **B** Quantification of relative protein levels in (**A**). Bars and error bars represent the mean values and SD from three independent experiments, respectively. The statistical significance of differences was determined using Student’s *t*-test. **p < 0.01. **C** Schematic representation of quantification of Flag-OASIS signal at the intact NE of main nuclei (NE), damaged NE of nuclear blebs (Bleb), and outside of these regions (Outside). Nuclear mask was generated using the signal of immunofluorescence staining of Lamin A/C. Note that Bleb is defined by chromatin herniation and partial or complete loss of Lamin A at the rim and that NE does not include the nuclear bleb for the quantification. **D** Immunofluorescence staining analysis of Flag-OASIS-expressing shLMNB1 cells treated with DMSO (upper panels) and MG-132 (lower panels). **E** Quantification of the ratio of signal intensity of Bleb to NE and Bleb to outside in single cell. Box plot represents the medians and interquartile ranges with Tukey-style whiskers. Statistical significance of the differences was determined using Mann–Whitney’s *U*-test. ***p < 0.005. *n* = 13 DMSO and *n* = 14 MG-132 from two independent experiments. Scale bars: 5 μm.

Our previous work demonstrated that OASIS is degraded by ubiquitin-proteasome pathway under normal condition and is stabilized upon ER stress [23]. Thus, we asked whether OASIS is stabilized at damaged NE by escaping from proteasome-dependent degradation. Increased protein level of OASIS upon treatment with a proteasome inhibitor MG-132 in Flag-OASIS expressing shLMNB1 cells was confirmed by western blot (Fig. 3A, B). Further, immunofluorescence analysis of Flag-OASIS was performed to quantify the relative signal intensity of Flag-OASIS at nuclear blebs to outside regions and to intact NE (Fig. 3C). We observed the higher signal intensity at the nuclear blebs to those of outside regions and NE in control cells. In contrast, in MG-132 treated cell, those ratios were significantly reduced to nearly 1.0. (Fig. 3D, E). These results suggest that OASIS could specifically escape from proteasome-dependent degradation at the damaged NE.

### OASIS responds to various types of NE stress

**Fig. 4:**
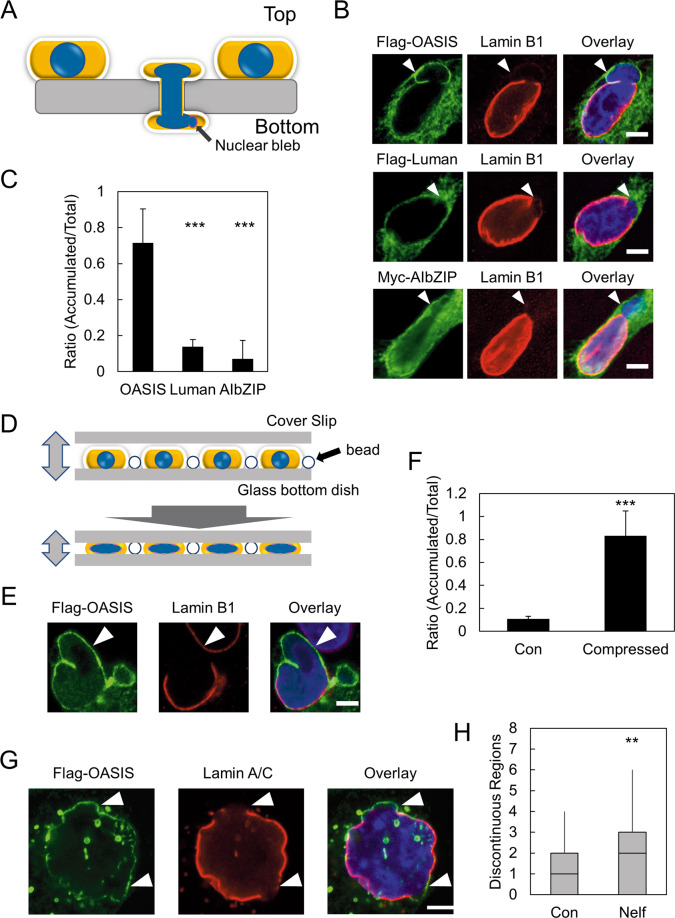
OASIS responds to various types of NE stress. **A** Schematic representation of constricted migration assay using Transwell. **B** Immunofluorescence staining analysis of Lamin B1 with Flag or Myc in Flag-OASIS-, Flag-Luman-, and Myc-AIBZIP-expressing HeLa cells on the bottom side of a Transwell. **C** Quantification of the ratio of the accumulation of OASIS, Luman, and AIbZIP on the nuclear blebs relative to total cells expressing the indicated proteins at the bottom side of a Transwell. Bars and error bars represent the mean values and SD from four independent experiments. Statistical significance of the differences was determined using Fisher’s exact test. ***p < 0.005. *n* = 37 OASIS, *n* = 33 Luman, *n* = 31 AIbZIP. **D** Schematic representation of cellular compression to induce NE rupture. **E** Immunofluorescence staining analysis of Lamin B1 and Flag-OASIS of the cells to which compressive force was applied. **F** Quantification of the ratio of the accumulation of OASIS to the regions where Lamin B1 was depleted in control cells (Con) and compressed cells (Compressed). Bars and error bars represent the mean values and SD from three independent experiments. Statistical significance of the differences was determined using Fisher’s exact test. ***p < 0.005. *n* = 36 Con, *n* = 41 Compressed. **G** Immunofluorescence staining analysis of Lamin A/C in Flag-OASIS-expressing HeLa cells treated with Nelfinavir. Arrowheads indicate the regions where Lamin A/C reveals discontinuous localization and OASIS accumulates. **H** Quantification of the number of regions where Lamin A/C is discontinuously localized in Flag-OASIS-expressing cells treated with DMSO (Con) and Nelfinavir (Nelf). Box plot represents the medians and interquartile ranges with Tukey-style whiskers. Statistical significance of the differences was determined using Mann–Whitney’s *U*-test. ***p < 0.005. *n* = 59 Con and *n* = 70 Nelf from three independent experiments. Scale bars: 5 μm.

To test whether OASIS also accumulates at damaged NE induced by different types of NE stress, we applied additional models of NE stress. Transwell migration assay can recapitulate the cell migration under constricted environments such as packed tissues or capillaries. As cells migrate through the porous membrane filter device with a small pore size such as Transwell, nuclear blebs are formed at the leading edge of the nucleus on the bottom side (Fig. 4A) [9, 10]. These nuclear blebs are devoid of Lamin B1 and are prone to being ruptured [24]. HeLa cells were transfected with the expression vectors of Flag-OASIS, Flag-Luman, and Myc-AIbZIP, and then plated on the top side of the Transwell. In 80% of cells expressing Flag-OASIS on the bottom side of the Transwell, Flag-OASIS accumulates at the periphery of nuclear blebs where Lamin B1 was depleted (Fig. 4B, C). In contrast, Flag-Luman and Myc-AIbZIP hardly accumulated on these nuclear blebs (Fig. 4B, C).

A recent study established that compressive force immediately induces NE rupture, thus can be used to monitor acute NE stress response (Fig. 4D) [13]. We transfected the expression vector of Flag-OASIS into HeLa cells and applied compressive force to these cells. Then, we observed the localization of Flag-OASIS and Lamin B1. We noticed that nuclei were severely deformed and Lamin B1 was depleted from extended regions of NE in the cells to which compressive force had been applied (Fig. 4E). Flag-OASIS accumulated on the damaged NE regions where Lamin B1 was depleted in ~80% of cells expressing Flag-OASIS after compression (Fig. 4E, F). These results indicate that OASIS accumulates on the damaged NE where Lamin B1 is depleted in response to acute NE stress.

It has been shown that the clinically used HIV protease inhibitor Nelfinavir impairs the maturation of Lamin A and induces the loss of NE integrity [25]. To test whether OASIS responds to NE stress induced by Nelfinavir, Flag-OASIS was expressed in HeLa cells and those cells were treated with Nelfinavir. We could not observe significant alterations of nuclear bleb formation upon Nelfinavir treatment. Instead, we found the discontinuous localization of Lamin A/C, which is a common structural abnormality caused by various types of NE stress (Fig. 4G) [1, 7, 8]. The number of discontinuous regions of Lamin A/C was significantly increased twofold in the cells treated with Nelfinavir compared with the control cells (Fig. 4H). We also observed that OASIS accumulated on those regions where Lamin A/C were absent (Fig. 4G). In summary, OASIS accumulates on the damaged NE where Lamins are absent in response to various types of NE stress.

### Exploring factors that colocalize with OASIS at damaged NE

**Fig. 5:**
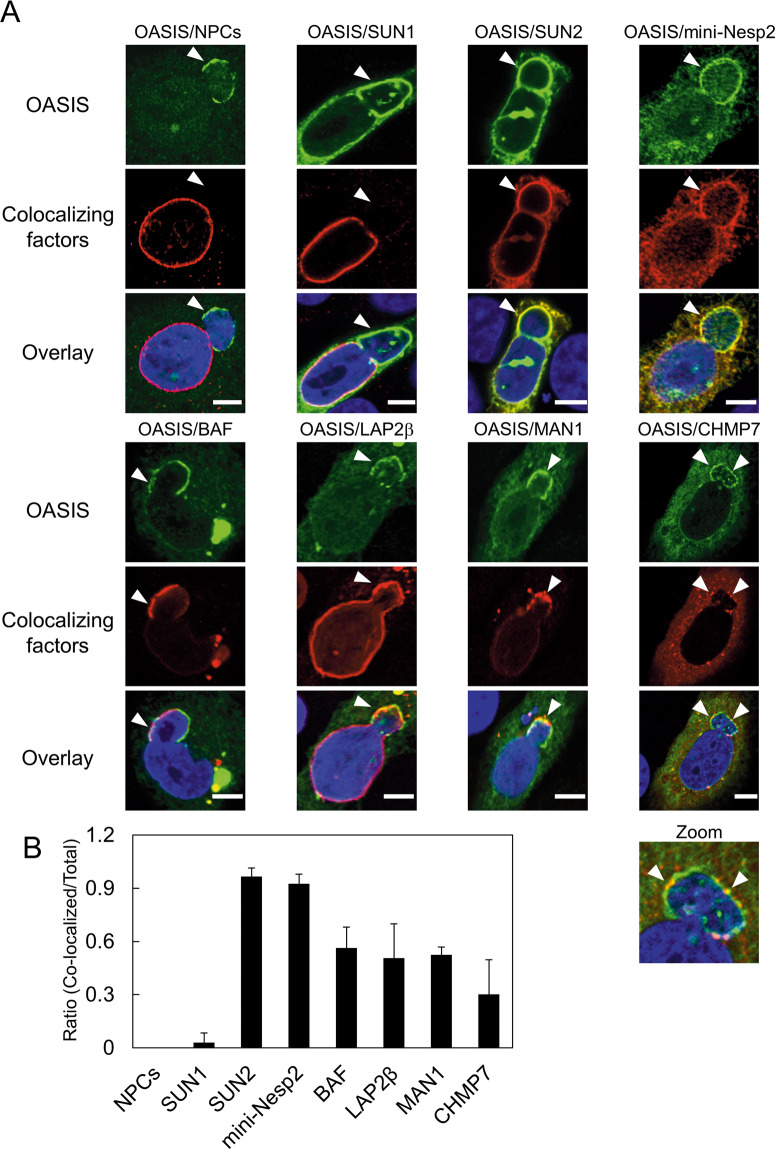
Exploring factors that colocalize with OASIS at damaged NE. **A** Immunofluorescent staining analysis of indicated factors with Flag-OASIS in shLMNB1 cells. Arrowheads indicate the nuclear blebs where OASIS accumulates. The following antibodies were used to visualize the indicated factors. NPCs: mAb414, a monoclonal antibody that recognizes multiple components of NPCs. SUN1 and SUN2: anti-Myc antibody, mini-Nesp2: anti-Nesprin-2 antibody. GFP-BAF, GFP-MAN1, and GFP-CHMP7: anti-GFP. LAP2β-GFP: anti-LAP2β. **B** Quantification of the ratio of colocalization of indicated factors with OASIS at the nuclear blebs. Bars and error bars represent the mean values and SD from at least three independent experiments. *n* = 23 NPCs, *n* = 32 SUN1, *n* = 32 SUN2, *n* = 27 mini-Nesp2, *n* = 25 BAF, *n* = 28 LAP2β, *n* = 31 MAN1, *n* = 30 CHMP7. Scale bars: 5 μm.

To identify the factors that colocalize with OASIS at damaged NE, we investigated the localization of a wide variety of proteins in shLMNB1 cells expressing Flag-OASIS. First, we investigated the localization of the nuclear pore complexes (NPCs) at the nuclear blebs where Flag-OASIS accumulates. NPCs were almost undetectable at the periphery of the nuclear blebs where Flag-OASIS accumulated. (Fig. 5A, B). The linker of nucleoskeleton and cytoskeleton (LINC) complexes are built from members of two conserved protein families: SUN domain and KASH domain proteins. SUN domain proteins are integral to inner nuclear membrane (INM), whereas KASH domain proteins reside in outer nuclear membrane (ONM) [26]. We investigated the localization of two major members of the SUN domain proteins: SUN1 and SUN2. The expression vectors of SUN1 and SUN2 tagged with Myc at their N-terminus were co-transfected with that of Flag-OASIS into shLMNB1. Interestingly, the colocalization of SUN2 with Flag-OASIS at the nuclear blebs was observed in 90% of cells, whereas that of SUN1 was found in only around 5% of cells (Fig. 5A, B). Additionally, we tested the localization of Nesprin-2, a major member of the KASH proteins in mammalian cells [26, 27]. We used a shorter artificial protein, mini-Nesprin-2, which contains N-terminal actin binding regions and a C-terminal KASH domain and is considered to recapitulate the localization pattern of the full-length form of Nesprin-2 [27]. The expression vector of monomeric red fluorescent protein mScarlet-I fused to mini-Nesprin-2 at its N-terminus (mSCI-mini-Neps2) was co-transfected with that of Flag-OASIS into shLMNB1 cells. Like SUN2, mini-Nesp2 revealed colocalization with Flag-OASIS at the nuclear blebs in 90% of the cells (Fig. 5A, B).

A subgroup of INM proteins named the LEM domain proteins share a conserved motif that is exposed to the nucleoplasm [28]. LEM domains of these proteins interact with BAF, a chromatin binding protein [29]. Recently, it has been demonstrated that BAF and LEM domain proteins target ESCRT III complex to ruptured NE sites, where it is proposed to assist in membrane resealing [14–17]. To test whether these factors colocalize with OASIS at nuclear blebs, the expression vectors of GFP-BAF, LAP2β-GFP, GFP-MAN1, and GFP-CHMP7 were co-transfected with that of Flag-OASIS into shLMNB1 cells. We found that GFP-BAF colocalized with OASIS at the nuclear blebs in ~50% of cells (Fig. 5A, B). Additionally, LAP2β-GFP and GFP-MAN1 also colocalized with OASIS at the nuclear blebs in around 50% of cells (Fig. 5A, B). Furthermore, GFP-CHMP7 signals were detected as the foci in a certain proportion of the cells (Fig. 5A, B). Few CHMP7 foci were observed in the nuclear blebs where OASIS accumulated in about 30% of cells (Fig. 5B).

### OASIS suppresses DNA damage induced by NE stress and maintains NE morphology

**Fig. 6:**
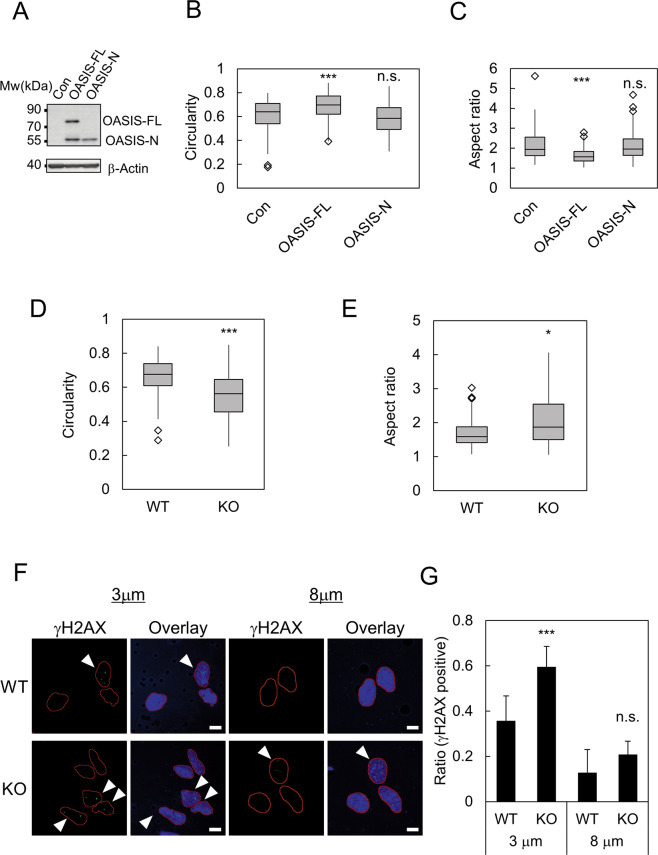
OASIS suppresses DNA damage induced by NE stress and maintains nuclear morphology. **A** Western blot analysis of U251-Con (Con) cells, U251-OASIS-FL (OASIS-FL) cells and U251-OASIS-N (OASIS-N) cells. The expression of Flag-OASIS-FL and Flag-OASIS-N was confirmed using anti-Flag antibody. β-Actin was used as a loading control. **B**, **C** Quantification of (**B**) nuclear circularity and (**C**) nuclear aspect ratio of U251-Con cells (Con), U251-OASIS-FL (OASIS-FL) cells, and U251-OASIS-N (OASIS-N) at the bottom side of a Transwell. Box plots represent the medians and interquartile ranges with Tukey-style whiskers. Statistical significance of the differences was determined using Mann–Whitney *U*-test. ***p < 0.005. n.s. *p* > 0.05. *n* = 64 Con, *n* = 45 OASIS-FL, and *n* = 43 OASIS-N from three independent experiments. **D**, **E** Quantification of (**D**) nuclear circularity and (**E**) nuclear aspect ratio of wild type (WT) and *Oasis* knockout (KO) primary astrocytes at the bottom side of a Transwell. Box plots represent the medians and interquartile ranges with Tukey-style whiskers. Statistical significance of the differences was determined using Mann–Whitney’s *U*-test. *p < 0.05. ***p < 0.005. *n* = 57 WT and KO from three independent experiments. **F** Immunofluorescent staining analysis of γH2AX in wild type (WT) and *Oasis* KO (KO) astrocyte at the bottom side of a 3 and 8 μm pore Transwell. The typical images of WT cells (upper panels) and KO cells (lower panels) were represented, respectively. Red lines indicated the boundaries of the nuclei. Arrow heads indicated γH2AX positive cells. **G** Quantification of the ratio of cells with γH2AX foci at the bottom side of a Transwell. Bars and error bars represent the mean values and SD from three (for 3 μm pore Transwell) or two (for 8 μm pore Transwell) independent experiments. Statistical significance of the differences was determined using Fisher’s exact test. ***p < 0.005. n.s. *p* > 0.05. *n* = 57 WT at 3 μm pore, *n* = 38 WT at 8 μm pore, *n* = 69 KO at 3 μm pore, *n* = 34 KO at 8 μm. Scale bars: 5 μm.

Next, we investigated the physiological significance of the NE stress response mediated by OASIS. We stably transfected the expression vector of Flag tagged full-length and N-terminus of OASIS or the empty vector into U251MG glioblastoma cells, which do not express endogenous OASIS. The resulting cell lines were designated as U251-OASIS-FL, U251-OASIS-N, and U251-Con, respectively. We confirmed the expression of full-length and N-terminus of OASIS by western blot (Fig. 6A). To test whether OASIS modulates cellular response to NE stress induced by constricted migration, these cells were plated on a Transwell. We noticed that the nuclei of U251-OASIS-FL cells were rounder than those of U251-Con and U251-OASIS-N at the bottom side of the Transwell. To evaluate the nuclear morphology of these cells under NE stress, we measured the circularity and the aspect ratio of the nucleus using DAPI staining. We found that nuclei of U251-OASIS-FL cells revealed a higher circularity than U251-Con at the bottom side of the Transwell (Fig. 6B). The nuclear aspect ratio of U251-OASIS-FL was significantly lower than that of U251-Con (Fig. 6C). The nuclear morphology of U251-OASIS-N cells were not altered compared with that of U251-Con. To further validate the role of endogenous OASIS, we used primary astrocytes derived from wild type (WT) and *Oasis* knockout (KO) mice [20]. The nuclei of the astrocytes derived from *Oasis* KO mice revealed lower circularity and higher aspect ratio compared with those of WT at the bottom side of Transwell, respectively (Fig. 6D, E). The role of OASIS in regulating nuclear morphology is likely not correlated with the alteration of cellular migration because neither U251-OASIS-FL nor *Oasis* KO astrocytes revealed significant difference in migration rate (Fig. S3A, B). It has been shown that the nuclear blebs induced by Transwell are prone to undergo persistent NE rupture and subsequently cause DNA damage [10, 24, 30]. Thus, we tested whether OASIS can suppress DNA damage that is induced by Transwell. The level of DNA damage at the bottom side of the Transwell was evaluated by γH2AX, a well-established marker of DNA double-strand breaks. KO astrocytes revealed a significantly higher ratio of γH2AX positive cells (60%) than WT astrocytes (36%) at the bottom side of the Transwell (Fig. 6F, G; 3 μm). To confirm that the role of OASIS is dependent on nuclear constriction, a Transwell with a larger pore size (8 μm), in which nuclei are not constricted, was used as a negative control [9]. As expected, the ratios of γH2AX positive cells at the bottom side of 8 μm pore Transwell was reduced than that of 3 μm pore Transwell in WT (13 %) as well as KO (21 %) (Fig. 6F, G; 8 μm). The difference of the ratios of γH2AX positive cells between WT and KO at the bottom side of the 8 μm Transwell was not statistically significant. Taken together, these results support that OASIS plays essential roles in the maintenance of NE morphology as well as the suppression of DNA damage induced by constricted migration.

## Discussion

In this study, we showed that an ER-resident transmembrane transcription factor, OASIS, accumulates on damaged regions of NE using several models of NE stress. Notably, Lamin proteins are depleted from the regions where OASIS accumulates, indicating their mutually exclusive localization pattern at the damaged NE (Figs. 1–4). Disruption of the nuclear lamina is a hallmark of NE damage caused by various types of NE stress [1, 31]. Recent studies have demonstrated that those structural abnormalities of nuclear lamina confer a predisposition to NE rupture [31]. Together with these findings, our results suggest that OASIS is a response factor against NE stress that disrupts the nuclear lamina, and could be used as a molecular marker for NE stress. It has been reported that transient transfection can induce defects in NE including the formation of nuclear blebs and the rupture of NE [32]. The overexpression of OASIS by transient transfection is unlikely to induce such abnormalities because the ratio of nuclear bleb positive cells in OASIS expressing cells is comparable to those of other OASIS family proteins (Fig. 2D).

The signals of the N-terminus and C-terminus of OASIS almost completely overlapped in the cells revealing nuclear blebs (Fig. 2B). Furthermore, a cleavage resistant OL-Chimera also accumulates at the nuclear blebs (Fig. S2B). Thus, it is likely that full-length OASIS accumulates at the damaged NE. Our functional analysis revealed that full-length but not N-terminus of OASIS suppresses nuclear deformation induced by Transwell (Fig. 6B, C). In addition, *Oasis* knockout primary cultured astrocytes revealed increased DNA damage only when they migrate through small size of pore (Fig. 6F, G). Previous studies have shown that NE rupture caused by constricted migration induces DNA damage and subsequently genome instability [6, 7, 10, 24, 30]. Taken together, these results suggest that OASIS functions in the maintenance of genome integrity by protecting against a broad spectrum of NE stresses. In addition, we showed that inhibition of proteasome attenuates the accumulation of OASIS at the nuclear blebs (Fig. 3D, E), suggesting that OASIS could escape from proteasome-mediated degradation at the nuclear blebs. This idea is supported by the absence of HRD1, a known major E3 ligase for OASIS, at the nuclear blebs (Fig. 1D) [23]. Further studies are required to clarify the overall dynamics of OASIS in response to NE stress. In particular, the visualization of endogenous OASIS by tagging with epitope tags or fluorescent proteins is highly desired because we could not investigate the endogenous localization of OASIS due to lack of antibodies that are compatible for immunofluorescent staining.

Our data demonstrated that NPCs and SUN1 are depleted from the nuclear blebs where OASIS accumulates, whereas SUN2 and Nesprin-2 colocalize with OASIS at those nuclear blebs (Fig. 5A, B). These results suggest that OASIS could play a certain role together with SUN2 and Nespirn-2 in the response to mechanical stress. Our results further revealed that BAF, LEM domain proteins, and ESCRT III component CHMP7 colocalize with OASIS at the nuclear blebs (Fig. 5B). Interestingly, the colocalization of NE repair factors with OASIS was clearly infrequent compared with that of SUN2 and Nesprin-2 (Fig. 5B). Such a difference can be accounted for by the dynamics of each factor in response to NE stress. SUN2 and Nesprin-2 might stably associate with the nuclear blebs where OASIS accumulates. In contrast, it is possible that NE repair factors such as BAF, LEM domain proteins, and CHMP7 are only recruited to the NE with severe damage, and dissociate from those regions when NE has been repaired [5, 6, 13, 14]. In this regard, the accumulation of OASIS at the nuclear blebs at a high frequency also indicates that OASIS could be retained on the damaged NE until Lamin proteins restore their normal organization at the nuclear blebs. Taking the obtained findings together, it is possible that OASIS plays a role in NE repair by co-localizing with BAF, LEM domain proteins, and CHMP7.

In summary, we demonstrated that (1) OASIS specifically accumulates on the damaged NE where Lamin proteins are depleted in response to various types of NE stress; (2) OASIS colocalizes with the components of the LINC complex and the factors involved in NE repair; and (3) OASIS suppresses DNA damage induced by NE rupture and maintains the nuclear morphology under NE stress. These findings suggest that OASIS is a key molecule that responds to NE stress and suppresses nuclear deformation as well as DNA damage caused by cellular migration under constricted environments.

## Materials and Methods

### Mice

The *Oasis*−/− (KO) mice (C57BL/6) were previously established in our laboratory [20]. For comparing wild type (WT) and *Oasis* KO mice, littermates derived from the mating of *Oasis*+/− mice were used. The experimental procedures and housing conditions for animals were approved by the Committee of Animal Experimentation, Hiroshima University.

### Cell culture, chemical treatment, and plasmids

HeLa cells and U2OS cells were maintained in Dulbecco’s Modified Eagle’s Medium (Gibco, Rockville, MD, USA) supplemented with 10% (v/v) heat-inactivated fetal bovine serum and penicillin/streptomycin at 37 °C in a 5% CO_2_, 95% humidified air atmosphere. U251MG cells were maintained under the same conditions except that Eagle’s Minimum Essential Medium (Gibco) was used as the basal medium. The shRNA expression vector for *LMNB*1 (pLKO.1 shRNA-LMNB1.71 puro, SHCLND-NM_005573, Sigma-Aldrich, St. Louis, MO, USA) and empty vector pLKO.1 were used for the production of lentiviruses. HeLa cells were infected with the produced lentiviruses and stable cell lines were established by puromycin selection at 3 μg/ml. The expression vector of Flag-tagged full-length (U251-OASIS-FL) and N-terminus (U251-OASIS-N) of OASIS and empty vector (U251-Con) were linearized, transfected into U251MG cells, and stable cell lines were established by puromycin selection at 2 μg/ml. Primary astrocytes were derived from WT and Oasis KO mice and maintained as described previously [33]. HeLa cells were treated with 40 μM Nelfinavir (TCI, Tokyo, Japan) for 4 h. shLMNB1 cells were treated with 10 μM MG-132 (Peptide Institute, Osaka, Japan) for 12 h.

A rapidly maturating mutant of mVenus, mVenusQ69M [34], was generated by site directed mutagenesis using mVenus-N, a kind gift from Michael Davidson and Atsushi Miyawaki (Addgene plasmid # 54640; http://n2t.net/addgene:54640) [35], and the following primer set: 5′-GGGCTACGGCCTGATGTGCTTCGCCCGC-3′ (sense) and 5′- GCGGGCGAAGCACATCAGGCCGTAGCCC-3′ (antisense). The obtained cDNA was cloned into pEGFP-C1 (Clontech, Mountain View, CA, USA) to replace EGFP by mVenus at *NheI*-*BsrGI* sites (pmVenus-C1). The expression vector of mVenus fused to human OASIS at its N-terminus (mVenus-OASIS) was constructed by PCR amplification of the cDNA of OASIS using the following primer sets and was cloned into pmVenus-C1 at *XhoI*-*KpnI* sites. 5′-CTGAACTCGAGGTATGGACGCCGTCTTGGAAC-3′ (OASIS-forward), 5′-TCGTAGGTACCCTAGGAGAGTTTGATGGTGG-3′ (OASIS-reverse). The expression vectors of GFP-MAN1 and GFP-CHMP7 were constructed by PCR using the following primer sets and were cloned into pEGFP-C1 at *BglII*-*SalI* sites. 5′-GAAGATCTGCGGCGGCAGCAGCTTCGGC-3′ (MAN1-forward), 5′-AACGCGTCGACTCAGGAACTTCCTTGAGAATTGGTTAGG-3′ (MAN1-reverse), and 5′-GAAGATCTTGGTCCCCGGAGCGGGAGGCCGAG-3′ (CHMP7-forward), 5′-AACGCGTCGACCTACAATGGCTTTAGAGTCGGTTCC-3′ (CHMP7-reverse). The mScarlet-I (mSCI) expression vector (pmSCI-C1) was constructed by *AgeI*-*BsrGI* digestion using the plasmid containing cDNA of mSCI, a gift from Dorus Gadella (Addgene plasmid # 98831; http://n2t.net/addgene:98831) [36]. The expression vector of mSCI fused to mini-Nesprin2 at its N-terminal (mSCI-mini-Nesp2) was constructed by two-step cloning. First, the C-terminal region including the KASH domain were cloned into pmSCI-C1 vector by PCR using the plasmid containing cDNA of Nesprin-2α kindly gifted by Catherine Shanahan and the primer set below with *XhoI-EcoRI* site [37]. 5′-ATCCTCGAGCTGGTGGCAAAGAAGGCCCGC-3′ (mini-Nesp2-C-forward) and 5′-ATGCGAATTCTGTGGGGGGTGGCCCATTGG-3′ (mini-Nesp2-C-reverse). Then the N-terminal region including actin binding domains were cloned into the resulting vector by PCR using the primer set below with *BsrGI* site. 5′-GAGCTGTACAAGTCTAGTCCTGAGCTTCCCACCGAAGATG-3′ (mini-Nesp2-N-forward), 5′-GCCTGTACAAATGAAATTCTAGAAGTAGAATAAATTTTTTCTCCAAAATG-3′ (mini-Neps2-N-reverse). Expression vectors for other epitope-tagged OASIS, Luman, and AIbZIP had been constructed as previously described [38, 39]. Expression vectors for Myc-SUN1 (pCINeo myc-mSUN1) and Myc-SUN2 (pL61CS myc-hSUN2) were kindly gifted by Sue Shackleton [37]. The expression vectors for EGFP-BAF (Addgene plasmid # 101772; http://n2t.net/addgene:101772) and LAP2β-AcGFP (Addgene plasmid # 62044; http://n2t.net/addgene:62044) were gifts from Daniel Gerlich and Eric Schirmer, respectively [40, 41]. Flag-OL-Chimera (amino acids 376–520 of OASIS was replaced with of amino acids 241–379 of Luman) was constructed by megaprimer PCR using primer sets below. 5′-CACGAGGACCAGAACACAGGTGCCGGTCTGCGTGGCTG-3′ (OASIS-N-Luman), 5′-GCGGCCGCCACCATGGACTACAAGGACGAC-3′ (NotI-Flag-Fwd), and 5′-AGTCTCGAGCTAACCTGAATACCTGCCC-3′ (Luman-Rev-XhoI). Transfection of expression vectors was performed using Avalanche-Everyday Transfection Reagent (EZBiosystems, College Park, MD, USA), in accordance with the manufacturer’s protocols.

### Protein preparation and western blotting

Proteins were extracted from the indicated cells in a cell lysis buffer containing 50 mM Tris-HCl (pH 7.5), 150 mM NaCl, 1% SDS, and Protease inhibitor cocktail Set V (Wako, Tokyo, Japan). The lysates were briefly sonicated. After centrifugation at 16,000x*g* for 5 min, the protein concentrations of the supernatants were determined using a bicinchoninic acid assay (BCA) kit (Thermo Fisher Scientific, Waltham, MA, USA). Equal amounts of proteins (10 μg) were used for sodium dodecyl sulfate–polyacrylamide gel electrophoresis (SDS-PAGE). For immunoblotting, the following antibodies were used: anti‐β‐actin (1:2000; MAB1501, Millipore, Burlington, MA, USA), anti‐Lamin B1 (1:2000; ab16048, Abcam, Cambridge, UK), and anti-OASIS monoclonal antibody produced with a hybridoma as described previously (1:1000) [21].

### Immunofluorescence staining and image analysis

The cells were grown on human fibronectin-coated coverslips or glass-bottom dishes (Matsunami Glass, Osaka, Japan) and fixed in 4% paraformaldehyde for 10 min. After the fixation, the cells were permeabilized in 0.5% Triton‐X 100 for 3 min, followed by blocking with 5% normal goat serum for 60 min. The following antibodies were used as the primary antibodies: anti-KDEL (1:250; Enzo Life Sciences, Farmingdale, NY, USA), anti-Lamin A/C (1:1000; SAB4200236, Sigma-Aldrich, St. Louis, MO, USA), anti‐HRD1 (1:250; #14773, Cell Signaling Technology, Beverly, MA, USA), anti‐Flag M2 (1:250; F1804, Sigma-Aldrich), anti-DYKDDDDK (1:250; #14793, Cell Signaling Technology), anti‐HA (1:250; #2367 and #3724, Cell Signaling Technology), anti-Myc (1:250; M192-3 and #562 MBL Nagoya, Japan), anti‐Lamin B1 (1:1000; Abcam), mAB414 (1:100; Biolegend, #902901, San Diego, CA, USA), anti‐GFP (1:250; #598, MBL), anti-Nesprin-2 (1:100; MABC86, Millipore), anti-LAP2β antibodies (1:250; #611000, BD Biosciences, San Jose, CA, USA), and anti-γH2AX antibodies (1:1000; #05–636, Millipore). Incubation with primary antibodies was performed at 4° overnight. For secondary antibodies, goat anti-mouse or -rabbit IgG F(ab′)2 fragments conjugated with Alexa 488 or 568 were used (Thermo Scientific). Confocal fluorescent images were acquired by FV1000D (Olympus, Tokyo, Japan) or LSM780 (Carl Zeiss, Jena, Germany). Image processing and image analysis were performed by ImageJ (National Institutes of Health, Rockville, MD, USA). For quantification of signal intensity of Flag-OASIS, nuclear masks were generated with the signal of Lamin A/C staining. The ROIs for damaged NE of nuclear blebs and intact NE of main nuclei, of which width was set at 0.39 μm, were generated by using the functions of Dilate, Erode, and Image Calculator. The ROIs for 0.39 μm outside of these regions were generated using the functions of Dilate and Image Calculator. For analysis of γH2AX positive cells, the cells that have at least one prominent γH2AX focus were judged as positive. The cells in which γH2AX signal is spread to entire nucleus were omitted from quantification.

### Constricted migration assay using Transwell

Constricted migration assay was performed using Transwell with a 3 μm pore size (Corning, Corning, NY, USA), as described previously [30]. HeLa cells were transfected with the indicated expression vectors and were cultured overnight. The next day, cells were detached using trypsin, replated on a Transwell, and cultured for an additional 36 h, followed by immunofluorescence staining. U251-Con cells, U251-OASIS-FL cells, U251-OASIS-N cells, and primary astrocytes were cultured on a Transwell for 24 h followed by further analysis.

### Cellular compression assay

Cellular compression assay was performed as described previously with minor modifications [14]. HeLa cells were transfected with Flag-OASIS expression vector and cultured overnight. The next day, the cells were replated on a *ϕ*27 mm glass-bottomed dish (Matsunami Glass) and cultured overnight again. Then the medium was supplemented with 3 μm polystyrene beads and an 18 × 18 mm cover glass attached with a silicon mold was placed on the glass bottom dish. The silicon mold was pressed by a 10 cm tissue culture dish and subjected to immunofluorescence staining as described above.

### 2D migration assay

U251-Con cells, U251-OASIS-FL cells, and primary astrocytes were plated in 2-well silicone chamber (ibidi, Gräfelfing, Germany) and cultured until they become confluent. Then silicon chambers were removed and the relative positions of leading edges was measured to calculate migration rates.

### Statistical analysis

To compare the expression level of Lamin B1 between control cells and shLMNB1, the expression level of Flag-OASIS between DMSO and MG-132 treated cells in shLMNB1 cells, the migration rates between WT and Oasis KO astrocytes, and the migration rates between U251-Con cells and U251-OASIS-FL cells, two tailed Student’s *t*‐test was used. To compare the rates of the indicated events between two samples, Fisher’s exact test was used. To compare numbers of discontinuous region of Lamin A/C, nuclear circularity, and nuclear aspect ratio, Mann–Whitney’s *U*-test was used. Microsoft Excel and R (version 4.0.2) were used via RStudio (version 1.3.1073) for statistical analysis [42, 43].

## Supplementary information

Figure S1 Localization mVenus-OASIS in U2OS cells.

Figure S2 Localization of OL-Chimera in shLMNB1 cells.

Figure S3 OASIS does not affect cellular migration.

## References

[1] Hatch E, Hetzer M (2014). Breaching the nuclear envelope in development and disease. J Cell Biol..

[2] de Noronha CM, Sherman MP, Lin HW, Cavrois MV, Moir RD, Goldman RD (2001). Dynamic disruptions in nuclear envelope architecture and integrity induced by HIV-1 Vpr. Science.

[3] De Vos WH, Houben F, Kamps M, Malhas A, Verheyen F, Cox J (2011). Repetitive disruptions of the nuclear envelope invoke temporary loss of cellular compartmentalization in laminopathies. Hum Mol Genet.

[4] Vargas JD, Hatch EM, Anderson DJ, Hetzer MW (2012). Transient nuclear envelope rupturing during interphase in human cancer cells. Nucleus.

[5] Denais CM, Gilbert RM, Isermann P, McGregor AL, te Lindert M, Weigelin B (2016). Nuclear envelope rupture and repair during cancer cell migration. Science.

[6] Raab M, Gentili M, de Belly H, Thiam HR, Vargas P, Jimenez AJ (2016). ESCRT III repairs nuclear envelope ruptures during cell migration to limit DNA damage and cell death. Science.

[7] Shimi T, Pfleghaar K, Kojima S, Pack C-G, Solovei I, Goldman AE (2008). The A- and B-type nuclear lamin networks: microdomains involved in chromatin organization and transcription. Genes Dev..

[8] Hatch EM, Hetzer MW (2016). Nuclear envelope rupture is induced by actin-based nucleus confinement. J. Cell Biol..

[9] Irianto J, Pfeifer CR, Bennett RR, Xia Y, Ivanovska IL, Liu AJ (2016). Nuclear constriction segregates mobile nuclear proteins away from chromatin. Mol Biol Cell.

[10] Irianto J, Xia Y, Pfeifer CR, Athirasala A, Ji J, Alvey C (2017). DNA damage follows repair factor depletion and portends genome variation in cancer cells after pore migration. Curr Biol..

[11] King MC, Lusk CP (2019). Loss of nuclear envelope integrity? No probLEM—BAF has it covered. J Cell Biol..

[12] Maciejowski J, Hatch EM (2020). Nuclear membrane rupture and its consequences. Annu Rev Cell Dev Biol..

[13] Halfmann CT, Sears RM, Katiyar A, Busselman BW, Aman LK, Zhang Q (2019). Repair of nuclear ruptures requires barrier-to-autointegration factor. J Cell Biol..

[14] Young AM, Gunn AL, Hatch EM (2020). BAF facilitates interphase nuclear membrane repair through recruitment of nuclear transmembrane proteins. Mol Biol Cell.

[15] Olmos Y, Perdrix-Rosell A, Carlton JG (2016). Membrane binding by CHMP7 coordinates ESCRT-III-dependent nuclear envelope reformation. Curr Biol..

[16] Gu M, LaJoie D, Chen OS, von Appen A, Ladinsky MS, Redd MJ (2017). LEM2 recruits CHMP7 for ESCRT-mediated nuclear envelope closure in fission yeast and human cells. Proc Natl Acad Sci USA.

[17] Honma Y, Kanazawa K, Mori T, Tanno Y, Tojo M, Kiyosawa H (1999). Identification of a novel gene, OASIS, which encodes for a putative CREB/ATF family transcription factor in the long-term cultured astrocytes and gliotic tissue. Brain Res Mol Brain Res.

[18] Omori Y, Imai J, Suzuki Y, Watanabe S, Tanigami A, Sugano S (2002). OASIS is a transcriptional activator of CREB/ATF family with a transmembrane domain. Biochem Biophys Res Commun..

[19] Kondo S, Murakami T, Tatsumi K, Ogata M, Kanemoto S, Otori K (2005). OASIS, a CREB/ATF-family member, modulates UPR signalling in astrocytes. Nat Cell Biol..

[20] Murakami T, Saito A, Hino S, Kondo S, Kanemoto S, Chihara K (2009). Signalling mediated by the endoplasmic reticulum stress transducer OASIS is involved in bone formation. Nat Cell Biol..

[21] Kondo S, Murakami T, Tatsumi K, Ogata M, Kanemoto S, Otori K (2005). OASIS, a CREB/ATF-family member, modulates UPR signalling in astrocytes. Nat Cell Biol..

[22] Asada R, Kanemoto S, Kondo S, Saito A, Imaizumi K (2011). The signalling from endoplasmic reticulum-resident bZIP transcription factors involved in diverse cellular physiology. J Biochem.

[23] Kondo S, Hino S-I, Saito A, Kanemoto S, Kawasaki N, Asada R (2012). Activation of OASIS family, ER stress transducers, is dependent on its stabilization. Cell Death Differ..

[24] Xia Y, Pfeifer CR, Zhu K, Irianto J, Liu D, Pannell K (2019). Rescue of DNA damage after constricted migration reveals a mechano-regulated threshold for cell cycle. J Cell Biol..

[25] Di Micco A, Frera G, Lugrin J, Jamilloux Y, Hsu E-T, Tardivel A (2016). AIM2 inflammasome is activated by pharmacological disruption of nuclear envelope integrity. Proc Natl Acad Sci USA.

[26] Rothballer A, Kutay U (2013). The diverse functional LINCs of the nuclear envelope to the cytoskeleton and chromatin. Chromosoma.

[27] Östlund C, Folker ES, Choi JC, Gomes ER, Gundersen GG, Worman HJ (2009). Dynamics and molecular interactions of linker of nucleoskeleton and cytoskeleton (LINC) complex proteins. J Cell Sci..

[28] Barton LJ, Soshnev AA, Geyer PK (2015). Networking in the nucleus: a spotlight on LEM-domain proteins. Curr Opin Cell Biol..

[29] Eriksson M, Brown WT, Gordon LB, Glynn MW, Singer J, Scott L (2003). Recurrent de novo point mutations in lamin A cause Hutchinson-Gilford progeria syndrome. Nature..

[30] Somech R, Shaklai S, Amariglio N, Rechavi G, Simon AJ (2005). Nuclear envelopathies—Raising the nuclear veil. Pediatr. Res..

[31] Robijns J, Houthaeve G, Braeckmans K, De Vos WH (2018). Loss of nuclear envelope integrity in aging and disease. Int Rev Cell Mol Biol..

[32] Lindenboim L, Sasson T, Worman HJ, Borner C, Stein R (2014). Cellular stress induces Bax-regulated nuclear bubble budding and rupture followed by nuclear protein release. Nucleus..

[33] Saito A, Kanemoto S, Kawasaki N, Asada R, Iwamoto H, Oki M (2012). Unfolded protein response, activated by OASIS family transcription factors, promotes astrocyte differentiation. Nat Commun..

[34] Cox RS, Dunlop MJ, Elowitz MB (2010). A synthetic three-color scaffold for monitoring genetic regulation and noise. J Biol Eng..

[35] Nagai T, Ibata K, Park ES, Kubota M, Mikoshiba K, Miyawaki A (2002). A variant of yellow fluorescent protein with fast and efficient maturation for cell-biological applications. Nat Biotechnol..

[36] Chertkova AO, Mastop M, Postma M, van Bommel N, van der NS, Batenburg KL, et al. Robust and bright genetically encoded fluorescent markers for highlighting structures and compartments in mammalian cells. bioRxiv [Preprint]. 2020 [cited 2020 Jan 13]:160374. Available from: 10.1101/160374

[37] Haque F, Mazzeo D, Patel JT, Smallwood DT, Ellis JA, Shanahan CM (2010). Mammalian SUN protein interaction networks at the inner nuclear membrane and their role in laminopathy disease processes. J Biol Chem..

[38] Cui X, Cui M, Asada R, Kanemoto S, Saito A, Matsuhisa K (2016). The androgen-induced protein AIbZIP facilitates proliferation of prostate cancer cells through downregulation of p21 expression. Sci. Rep..

[39] Kanemoto S, Kobayashi Y, Yamashita T, Miyamoto T, Cui M, Asada R (2015). Luman is involved in osteoclastogenesis through the regulation of DC-STAMP expression, stability and localization. J Cell Sci..

[40] Samwer M, Schneider MWG, Hoefler R, Schmalhorst PS, Jude JG, Zuber J (2017). DNA cross-bridging shapes a single nucleus from a set of mitotic chromosomes. Cell.

[41] Zuleger N, Kelly DA, Richardson AC, Kerr ARW, Goldberg MW, Goryachev AB (2011). System analysis shows distinct mechanisms and common principles of nuclear envelope protein dynamics. J Cell Biol..

[42] R Core Team. R: A language and environment for statistical computing. R Foundation for Statistical Computing, Vienna, Austria. 2020. Available from https://www.R-project.org/.

[43] RStudio Team. RStudio: Integrated Development for R. RStudio, PBC, Boston, MA. 2020. Available from http://www.rstudio.com/.

