# Supplementary material for: OASIS/CREB3L1 is a factor that responds to nuclear envelope stress
Source: Cell Death Discov. 2021 Jun 29;7:152. doi: 10.1038/s41420-021-00540-x (PMC8257603; doi:10.1038/s41420-021-00540-x)
Supplement: Supplementary file 1 — Figure S1 Localization mVenus-OASIS in U2OS cells. [file 41420_2021_540_MOESM1_ESM.pdf]

Figure S1

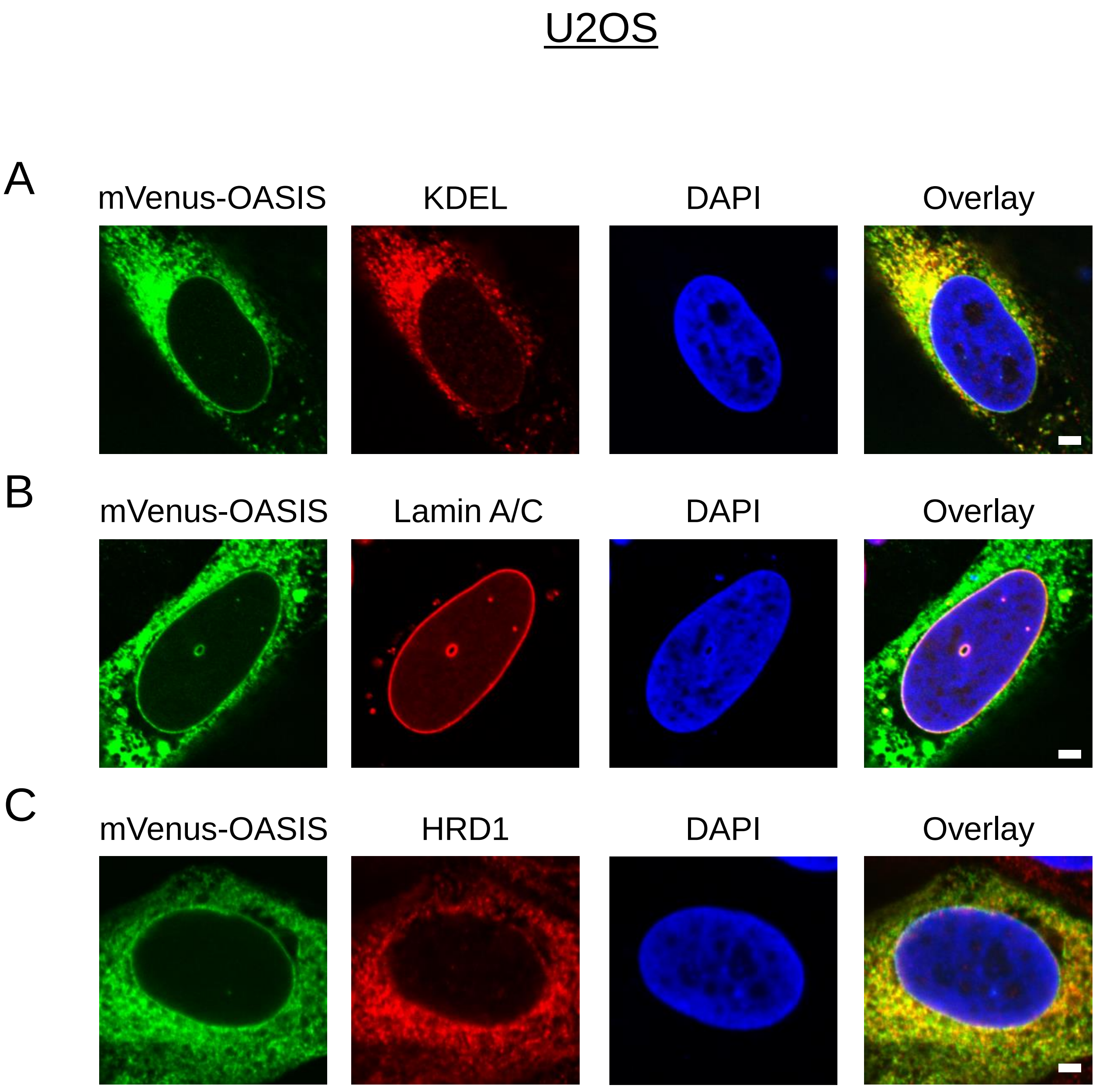

Fig. S1. Localization of mVenus-OASIS in U2OS cells. A, B, and C Immunofluorescence staining analysis of (A) KDEL, (B) Lamin A/C, and (C) HRD1 in U2OS cells expressing mVenus-OASIS. Scale bars: 5 μm.
