# Supplementary material for: OASIS/CREB3L1 is a factor that responds to nuclear envelope stress
Source: Cell Death Discov. 2021 Jun 29;7:152. doi: 10.1038/s41420-021-00540-x (PMC8257603; doi:10.1038/s41420-021-00540-x)
Supplement: Supplementary file 2 — Figure S2 Localization of OL-Chimera in shLMNB1 cells. [file 41420_2021_540_MOESM2_ESM.pdf]

Figure S2

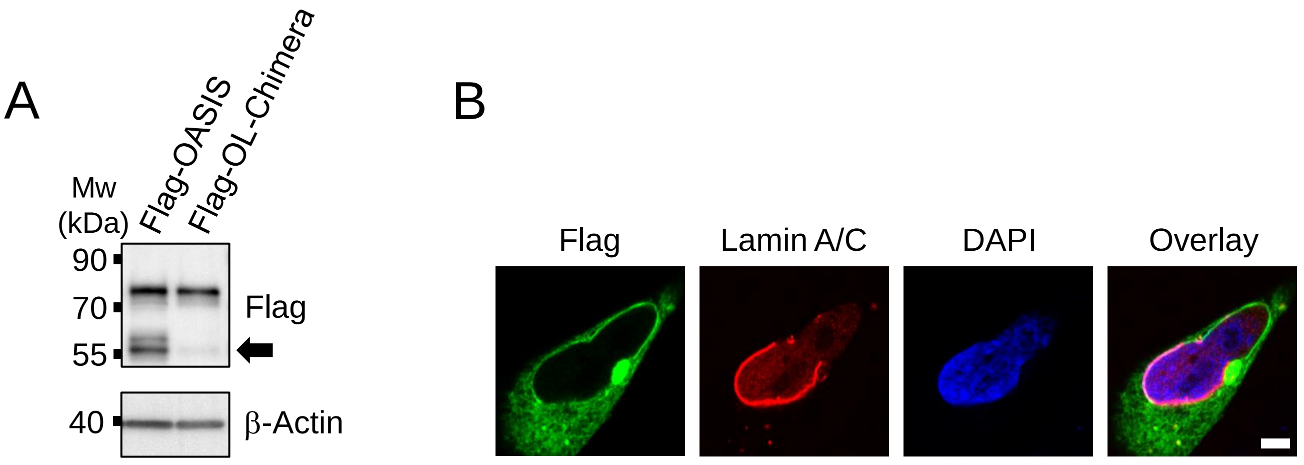

Fig. S2. Localization of Flag-OL-Chimera in shLMNB1 cells. A Western blot analysis of Flag-OL-Chimera. Arrow indicates N-terminal fragment produced by basal level cleavage of OASIS. β-Actin was used for a loading control. **B** Immunofluorescence staining analysis of Flag-OL-Chimera in shLMNB1 cells in which nuclear blebs formed. Scale bars: 5 μm.
