# Supplementary material for: OASIS/CREB3L1 is a factor that responds to nuclear envelope stress
Source: Cell Death Discov. 2021 Jun 29;7:152. doi: 10.1038/s41420-021-00540-x (PMC8257603; doi:10.1038/s41420-021-00540-x)
Supplement: Supplementary file 3 — Figure S3 OASIS does not affect cellular migration. [file 41420_2021_540_MOESM3_ESM.pdf]

Figure S3

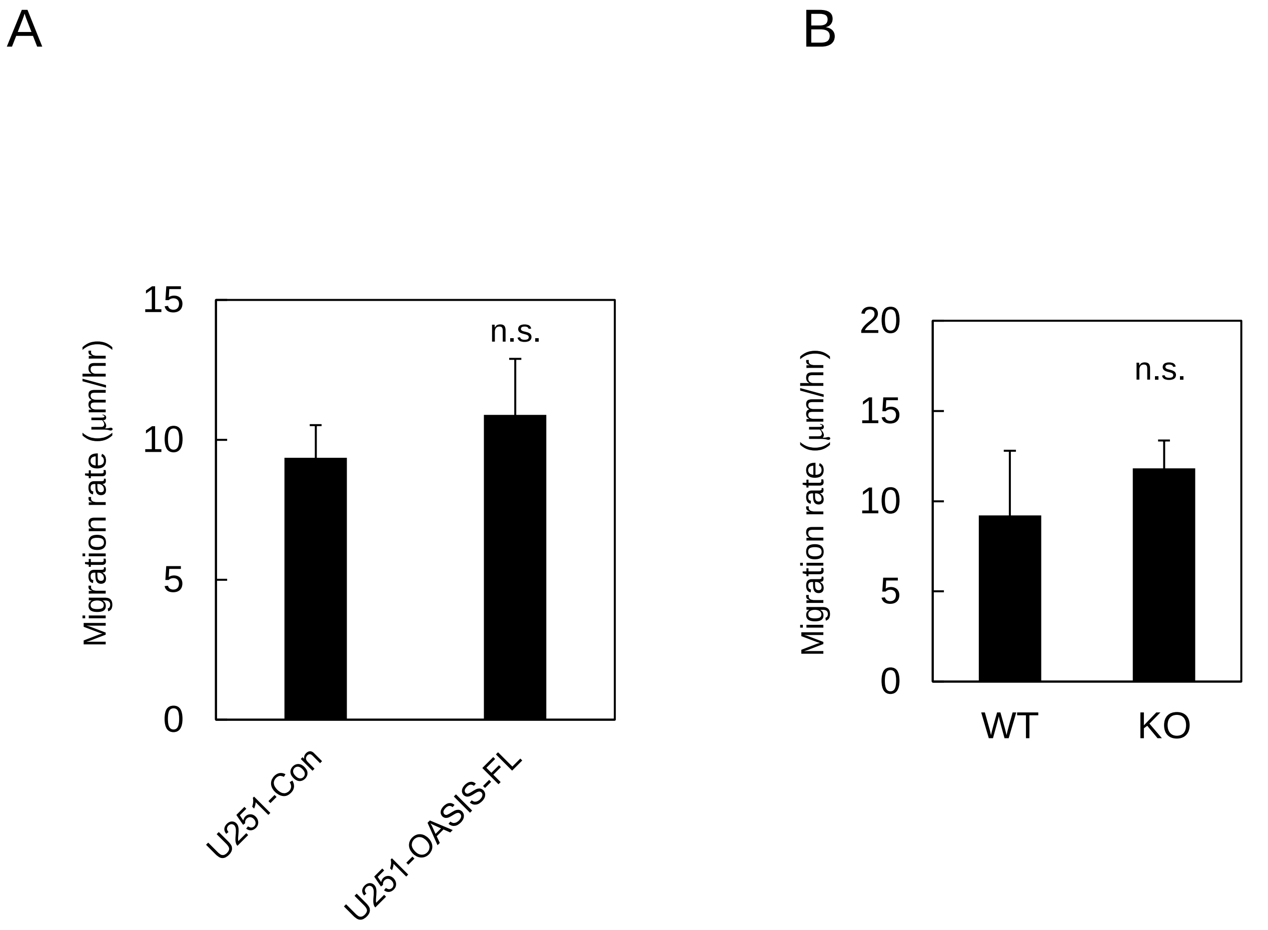

Fig. S3. OASIS does not affect cellular migration. A and **B** The quantification of the migration rate of (A) U251-Con and U251-OASIS-FL cells, and (B) WT and *Oasis* KO astrocytes. Bars and error bars represent the mean values and SD from two (U251) or three (astrocytes) independent experiments, respectively. The statistical significance of differences was determined using Student's *t*-test. n.s. $p>0.05$.
